# Uterine epithelial Gp130 orchestrates hormone response and epithelial remodeling for successful embryo attachment in mice

**DOI:** 10.1038/s41598-023-27859-y

**Published:** 2023-01-16

**Authors:** Takafumi Namiki, Jumpei Terakawa, Harumi Karakama, Michiko Noguchi, Hironobu Murakami, Yoshinori Hasegawa, Osamu Ohara, Takiko Daikoku, Junya Ito, Naomi Kashiwazaki

**Affiliations:** 1 Laboratory of Animal Reproduction, School of Veterinary Medicine, Azabu University, 1-17-71 Fuchinobe, Chuo-Ku, Sagamihara, Kanagawa 252-5201 Japan; 2 Graduate School of Veterinary Science, Azabu University, Sagamihara, Japan; 3 Laboratory of Toxicology, School of Veterinary Medicine, Azabu University, 1-17-71 Fuchinobe, Chuo-Ku, Sagamihara, Kanagawa 252-5201 Japan; 4 Laboratory of Theriogenology, School of Veterinary Medicine, Azabu University, Sagamihara, Japan; 5 Laboratory of Infectious Diseases, School of Veterinary Medicine, Azabu University, Sagamihara, Japan; 6 Department of Applied Genomics, Kazusa DNA Research Institute, Kisarazu, Japan; 7 Research Center for Experimental Modeling of Human Disease, Institute for Experimental Animals, Kanazawa University, Kanazawa, Japan; 8 Center for Human and Animal Symbiosis Science, Azabu University, Sagamihara, Japan; 9 Present Address: Department of Life Science Frontiers, Center for iPS Cell Research and Application (CiRA), Kyoto University, Kyoto, Japan

**Keywords:** Cell biology, Molecular medicine, Reproductive biology

## Abstract

Leukemia inhibitory factor (LIF) receptor, an interleukin 6 cytokine family signal transducer (Il6st, also known as Gp130) that is expressed in the uterine epithelium and stroma, has been recognized to play an essential role in embryo implantation. However, the molecular mechanism underlying Gp130-mediated LIF signaling in the uterine epithelium during embryo implantation has not been elucidated. In this study, we generated mice with uterine epithelium specific deletion of Gp130 (*Gp130* ecKO). *Gp130* ecKO females were infertile due to the failure of embryo attachment and decidualization. Histomorphological observation revealed that the endometrial shape and embryo position from *Gp130* ecKO were comparable to those of the control, and uterine epithelial cell proliferation, whose attenuation is essential for embryo implantation, was controlled in *Gp130* ecKO. Comprehensive gene expression analysis using RNA-seq indicates that epithelial Gp130 regulates the expression of estrogen- and progesterone-responsive genes in conjunction with immune response during embryo implantation. We also found that an epithelial remodeling factor, snail family transcriptional repressor 1 (Snai1), was markedly reduced in the pre-implantation uterus from *Gp130* ecKO. These results suggest that not only the suppression of uterine epithelial cell proliferation, but also Gp130-mediated epithelial remodeling is required for successful implantation in mice.

## Introduction

In mammals, embryo implantation is the first contact between mother and fetus^1^. Failure of implantation causes infertility, which is associated with abnormal endometrial receptivity^2^. During pregnancy, endometrial cells (including luminal epithelium, glandular epithelium, and stromal cells) cross-talk and undergo drastic morphological and/or functional changes^1^. These changes in the endometrium are tightly regulated by two steroid hormones, progesterone (P4) and 17β-estradiol (E2), which are produced mainly by the ovaries^3^. Both of these hormones act via their respective receptors and regulate their downstream target genes, leading to successful pregnancy^4^.

In mice, embryo implantation usually occurs at day 4 midnight of pregnancy (where day 1 is defined as the day when the vaginal plug is confirmed.) and is regulated by P4 and E2^3^. Implantation is a sequential process that includes embryo hatching, apposition, attachment, and invasion^2^. The shape of the uterine lumen changes from a jagged circular to a slit-like structure during peri-implantation, and the embryos are located on the anti-mesometrial side of the lumen, where the implantation chamber (also called the crypt) is formed^2^. A transient nidatory surge of E2 induces embryo attachment through leukemia inhibitory factor (LIF) signaling in concomitant with luteal-derived P4 action^1^. *Lif*-deficient females exhibit infertility due to the failure of embryo attachment^5^. While *Lif*-deficient mice are unable to support embryo implantation, their blastocysts implant normally when transplanted to pseudo-pregnant recipients^5^. It is widely accepted that LIF expression is facilitated by nidatory E2 in the glandular epithelium and that secreted LIF binds to its receptor, which is composed of LIF receptor (LIFR) and interleukin 6 cytokine family signal transducer (Il6st, also known as glycoprotein 130: Gp130), expressed in the luminal epithelium^6^. Ligand receptor complexes subsequently induce the phosphorylation of signal transducer and activator of transcription 3 (STAT3) and also activate target genes such as heparin-binding epidermal growth factor (EGF)-like growth factor (HB-EGF), which is essential for the recognition of the embryo via epidermal growth factor receptor receptor (EGFR/ErbB)^7^.

Although the detailed mechanism underlying LIF signaling is not fully understood, previous reports using tissue-specific gene knockout mice have demonstrated that a defect in LIF-mediated signaling is potentially the cause of embryo implantation failure (Supplementary Table 1). Uterine (whole uterus)-specific *Stat3*-deficient females derived by *Pgr*^*Cre*^ show implantation failure because of excessive estrogen responses, which preclude uterine remodeling to the receptive state for implantation^8,9^. Another recent study showed that uterine epithelial-specific *Lifr*-deficient females also exhibit a severely greater frequency of embryo implantation failure with no phosphorylation of STAT3, whereas stromal-specific *Lifr*-deficient females show no obvious phenotypes^10^. As for Gp130, uterine-specific gene knockout females exhibited infertility due to increased estrogen responsiveness and decreased progesterone responsiveness, leading to abnormally sustained luminal epithelial proliferation at the moment of embryo implantation^8^.

As mentioned above, LIF should bind to Gp130/LIFR heterodimer on the luminal epithelium; however, co-localization of Gp130 and LIFR is limited in the epithelium during embryo implantation^6^. The expression of *Lifr* is detected in both luminal and glandular epithelium before embryo implantation, but that of *Gp130* is dominantly detected in the glandular epithelium^8,11,12^. *Lif*, *Lifr*, and *Gp130* are all detectable in the uterine stroma during embryo implantation^6,11,12^, suggesting that stromal Gp130 might also be important. Moreover, Gp130 as a homodimer can bind other interleukin 6 (IL6) family cytokines such as IL-6 and IL-11, which are expressed in the uterus during early pregnancy^13,14^. Hence, although uterine-specific *Gp130*-deficient females show infertility, the role of epithelial Gp130 on embryo implantation has yet to be determined.

The aim of the present study was to clarify Gp130-mediated LIF signaling in the uterine epithelium. We confirmed that *Gp130* was dominantly expressed in the glandular epithelium, but not in the luminal epithelium, during embryo implantation. Nevertheless, uterine epithelial-specific *Gp130*-deficient (*Gp130* ecKO) females, driven by lactoferrin (*Ltf*)-iCre^15^, showed embryonic implantation failure. Epithelial cell proliferation and crypt formation were correctly regulated in *Gp130* ecKO, suggesting that, contrary to a previous report, the cause of infertility is distinct from uterine-specific *Gp130* knockout^8^. We also found that uterine epithelial remodeling toward embryo implantation was disrupted in *Gp130* ecKO. Thus, our results indicate that embryo implantation requires substantial changes in the properties of the uterine epithelium by Gp130-mediated signaling.

## Results

### Localization of Gp130 in the endometrium during early pregnancy

**Figure 1:**
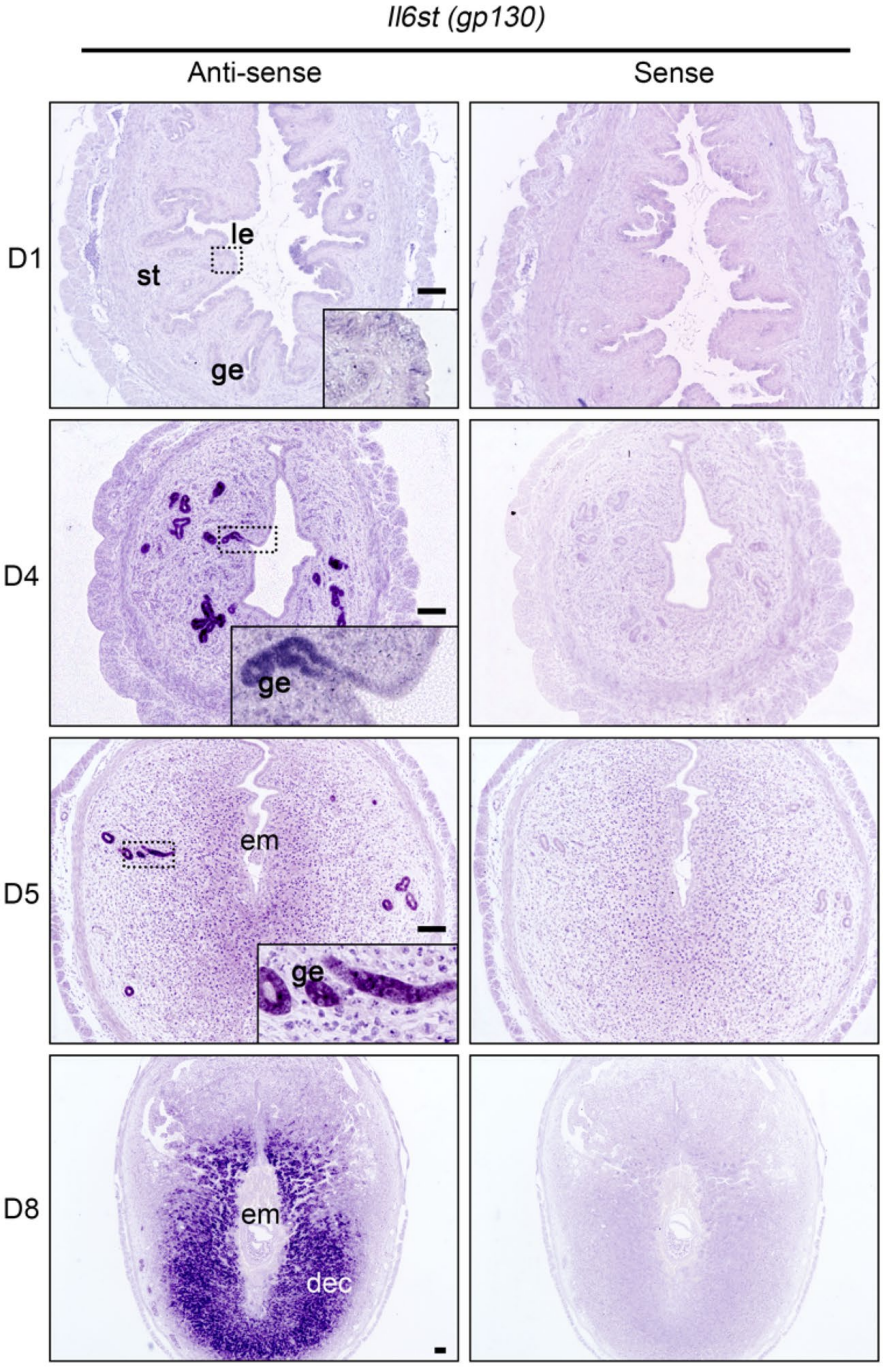
Spatiotemporal expression of *Il6st* (*Gp130*) mRNA in the mouse uterus during early pregnancy. Signals (dark purple) are detected by an anti-sense probe (left). The sense probe (right) shows the background signal. *Gp130* mRNA, which was almost undetectable at day 1 of pregnancy (D1), is expressed in the glandular epithelium (ge), but not in the luminal epithelium (le), before embryo implantation (D4), and subsequently is expressed in differentiating stromal cells and decidual cells (D5 and D8). Scale bar, 100 µm. *le* luminal epithelium, *ge* glandular epithelium, *st* stroma, *em* embryo, *dec* decidua.

We initially examined the expression and localization of *Gp130* by in situ hybridization (ISH). The expression of *Gp130* was low in day 1 uterine tissues after ovulation (Fig. 1). *Gp130* mRNA was increased in the glandular epithelium, but not in the luminal epithelium, on day 4 of pregnancy just before embryo attachment (Fig. 1). On day 5, just after embryo attachment, *Gp130* mRNA was expressed in glandular epithelial cells located on the edge and was slightly positive in the subepithelial stromal cells (Fig. 1). Differentiating stromal cells strongly expressed *Gp130* mRNA at day 8 (Fig. 1), and decidualized stromal cells located on the maternal border in the placenta subsequently sustained *Gp130* mRNA at day10 (Supplemental Fig. 1).

### Uterine epithelial-specific Gp130 deletion leads to infertility due to embryo attachment failure

**Figure 2:**
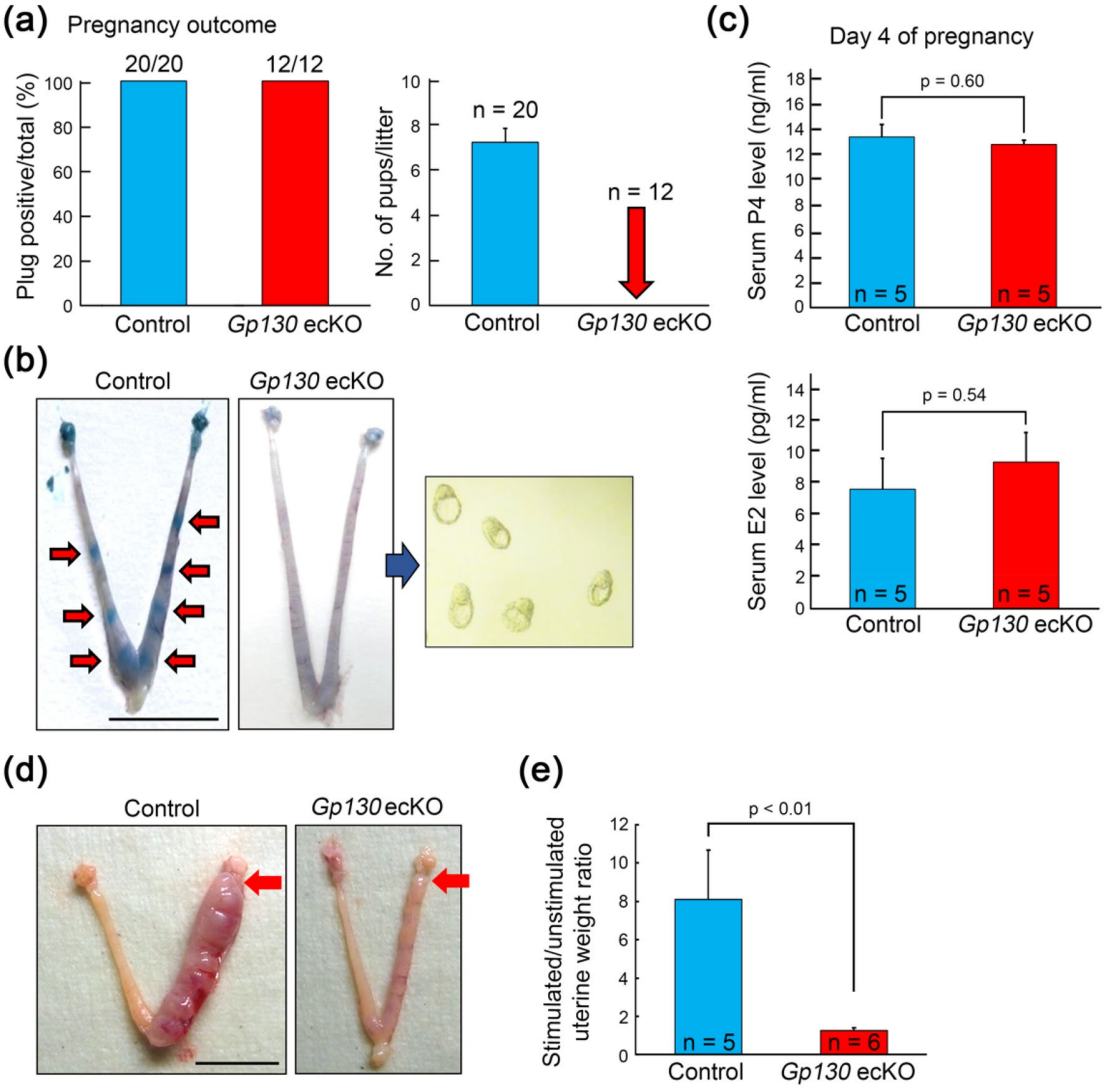
Uterine epithelial-specific deletion of *Gp130* confers female infertility. (**a**) Pregnancy outcomes in the control and epithelial-specific deletion of *Gp130* (*Gp130* ecKO) females. *Gp130* ecKO females are completely infertile even though they all mated with a wildtype male. (**b**) Representative uteri from control and *Gp130* ecKO females on D5. Implantation sites (red arrows), which are visualized by a blue dye injection in the control, are not seen in *Gp130* ecKO. Unimplanted blastocysts were recovered from *Gp130* ecKO uteri. (**c**) Serum levels of progesterone (P4) and 17β-estradiol (E2) at D4. (**d**) Representative pictures showing the gross morphology of artificial decidualization (4 days after oil injection) from control and *Gp130* ecKO. Red arrow indicates the site of sesame oil injection. (**e**) The ratio between the wet weight of the oil-injected (stimulated) horn and that of the unstimulated horn was calculated. Scale bar, 1 cm.

To assess whether the deletion of Gp130 in the uterine epithelium affects pregnancy outcome in mice, we crossed the control and *Gp130* ecKO females with sexually mature wildtype males to confirm their fertility. Although vaginal plugs were found in all individuals from both the control and *Gp130* ecKO (Fig. 2a), *Gp130* ecKO females were unable to produce any pups, whereas the average number of pups delivered in the control was 7.2 ± 0.6 (Fig. 2a). In *Gp130* ecKO mice, no implantation site was observed at day 5 (Fig. 2b), and hatched and expanded blastocysts were recovered from the uterine horns (Fig. 2b).

We measured the serum levels of P4 and E2 and found that they were comparable between the control and *Gp130* ecKO at day 4 of pregnancy (Fig. 2c). The control females formed deciduoma after artificial decidualization, but the reaction was absent in *Gp130* ecKO (Fig. 2d,e).

**Figure 3:**
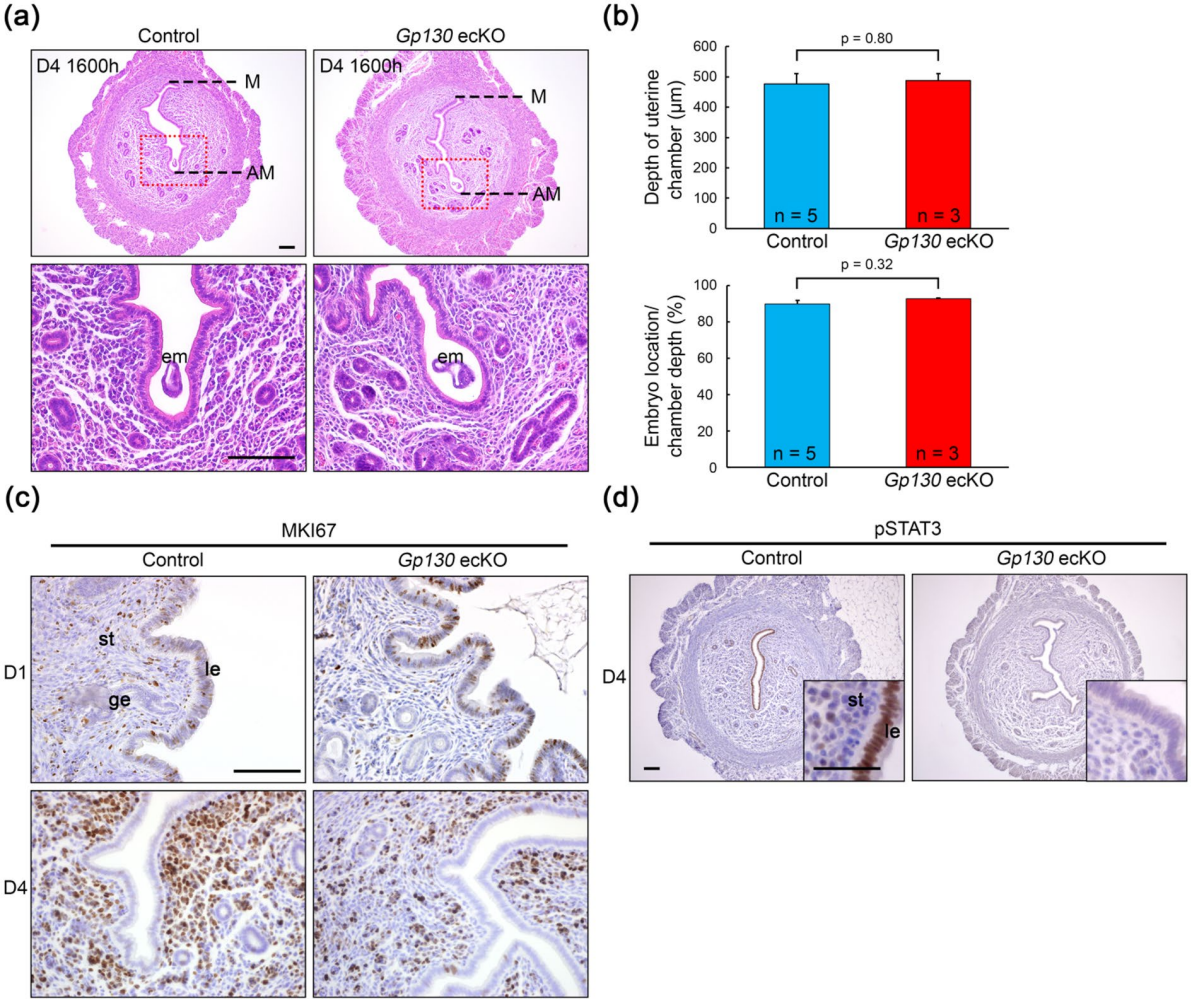
Histological and histochemical characterization in *Gp130* ecKO uterus before embryo implantation. (**a**) Representative images of uterine cross section of pre-implantation site on D4 (1600 h) from control and *Gp130* ecKO. The depth of the uterine chamber (M-AM distance) is comparable between control and *Gp130* ecKO (**b**, upper panel), and embryos are located in the deep antimesometrial side of the uterine chamber in both groups (**b**, lower panel). (**c**) Cell proliferation status detected by immunostaining of MKI67 at D1 (1000 h) and D4 (1600 h). (**d**) Immunostaining of phosphorylated STAT3 (pSTAT3) in pre-implantation uterus (D4, 1600 h). pSTAT3 is not detectable in the uterine epithelium from *Gp130* ecKO. Scale bar, 100 µm (**a**,**c**, and **d** with lower magnification) and 50 µm (**d** with higher magnification). *le* luminal epithelium, *ge* glandular epithelium, *st* stroma, *em* embryo, *M* mesometrial side, *AM* antimesometrial side.

Histological observation of day 4 uterine tissues showed that both the control and *Gp130* ecKO exhibited slit-like structures, which are characteristic of the uterus before embryo attachment (Fig. 3a). The uterine area was comparable between the control and *Gp130* ecKO (Supplementary Fig. 2). Blastocysts were placed in the antimesometrial side of the crypt in *Gp130* ecKO as well as in the control (Fig. 3a,b), meaning that the implantation chamber was correctly formed in *Gp130* ecKO. In a previous study, mice genetically deficient in whole uterine Gp130 showed embryo implantation failure, and the cause of their infertility was found to be the loss of control over endometrial epithelial cell proliferation due to increased E2 reactivity^8^. Therefore, we checked whether epithelial cell proliferation had ceased by day 4 in *Gp130* ecKO by immunostaining the MKI67 (Fig. 3c). Epithelial cell proliferation was observed at day 1 but was suspended at day 4 in both the control and *Gp130* ecKO (Fig. 3c and Supplementary Fig. 3). LIF administration induced the phosphorylation of STAT3 (pSTAT3) in the pre-receptive uterine epithelium^16^. pSTAT3 was observed in the nuclei of the uterine epithelium in the control at day 4, but no pSTAT3 was observed in *Gp130* ecKO (Fig. 3d).

### Loss of uterine epithelial Gp130 results in abnormal expression of hormonal responsive factors

**Figure 4:**
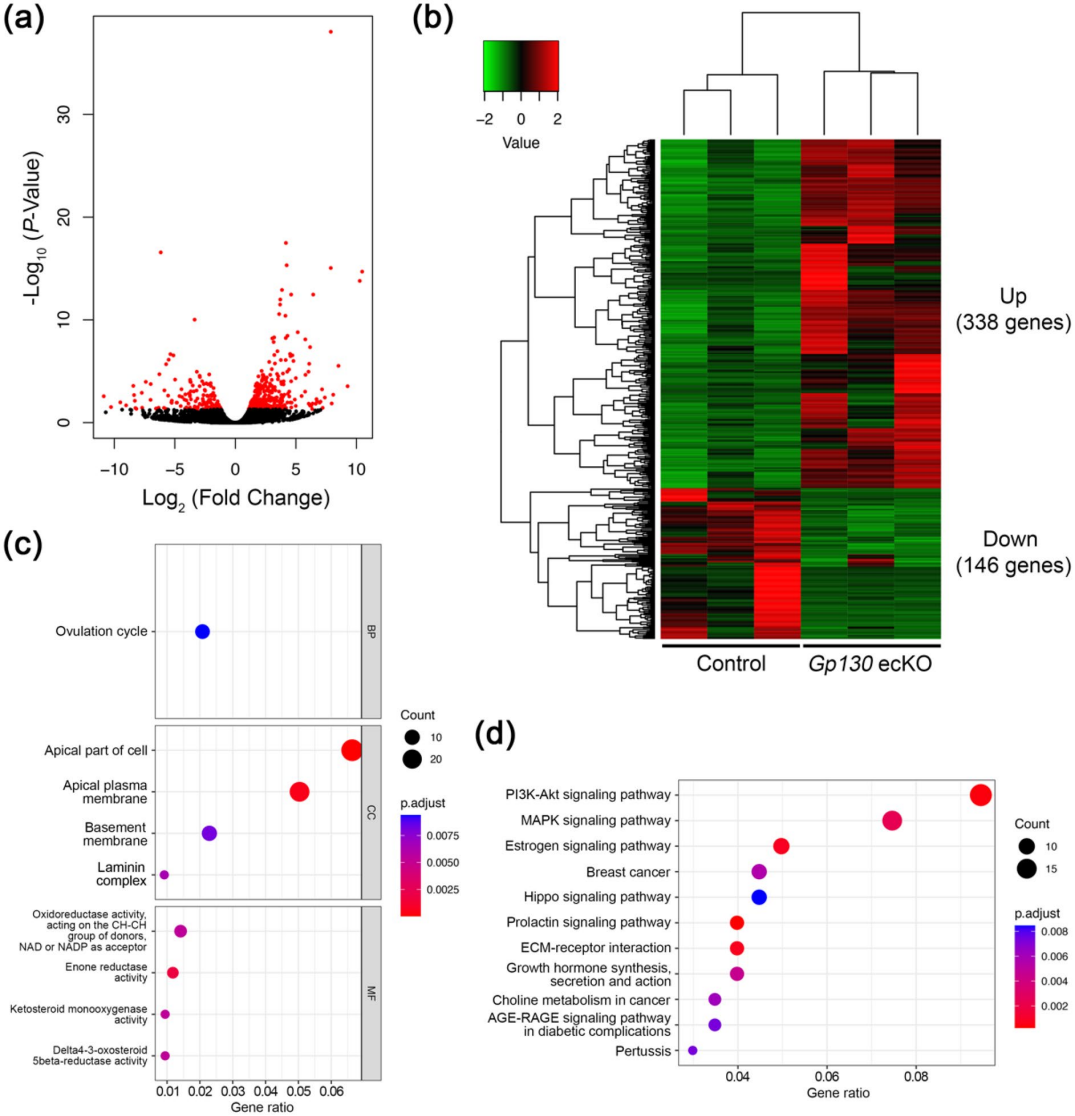
Comprehensive gene expression analysis between control and *Gp130* ecKO uteri before embryo implantation. (**a**) Volcano plot displaying differentially expressed genes whose expression levels were altered by a two-fold increase or decrease in *Gp130* ecKO compared to the control (marked in red, FDR < 0.05). (**b**) Heatmap of differentially expressed genes. 338 genes are upregulated and 146 genes are down regulated in *Gp130* ecKO. Heatmap was generated by the gplots v3.1.3 package in R v4.2.1 (URL https://www.R-project.org/). (**c**) Systematic characteristics of differentially expressed genes in *Gp130* ecKO uteri compared to the control by GO analysis. *BP* biological process, *CC* cellular component, *MF* molecular function. (**d**) Selected significant pathway altered in *Gp130* ecKO uteri.

To investigate the molecular mechanism underlying embryo attachment failure in *Gp130* ecKO, we conducted a comprehensive gene expression analysis on day 4 uteri of the control and *Gp130* ecKO by RNA-seq. Differential gene expression analysis between the control and *Gp130* ecKO identified 484 differentially expressed genes (> two-fold change, false discovery rate (FDR) < 0.05, Fig. 4a), in which 338 genes were upregulated and 146 genes were down regulated in *Gp130* ecKO compared to the control (Fig. 4b and Supplementary Table 2). Upregulated genes included estrogen receptor α (*Esr1*) and E2 responsive genes such as lactotransferrin (*Ltf*)^17^ and insulin receptor substrate 1 (*Irs1*)^18^. On the other hand, down regulated genes in *Gp130* ecKO covered P4-responsive genes, including arachidonate 15-lipoxygenase (*Alox15*)^17^ and amphiregulin (*Areg*)^19^, and suppressor of cytokine signaling 3 (*Socs3*), which is stimulated by STAT3-dependent pathway^20^. Notably, genes regulated by LIF, cochlin (*Coch*)^21^ and insulin-like growth factor binding protein 3 (*Igfbp3*)^22^, were significantly down regulated in *Gp130* ecKO. In addition, gene expression associated with uterine receptivity was disrupted in *Gp130* ecKO: epiregulin (*Ereg*, down regulated)^23^, early growth response 1 (*Egr1*, down regulated)^24,25^ and serum/glucocorticoid regulated kinase 1 (*Sgk1*, up-regulated)^26^. The results of the Gene ontology (GO) analysis revealed that genes related to apical part of cell and apical plasma membrane were enriched in differentially expressed genes (Fig. 4c and Supplementary Fig. 4). Pathway analysis showed that differentially expressed genes were associated with several pathways, including phosphatidylinositol 3-kinase/AKT (PI3K-Akt), mitogen-activated protein kinase (MAPK) and E2 signaling (Fig. 4d).

**Figure 5:**
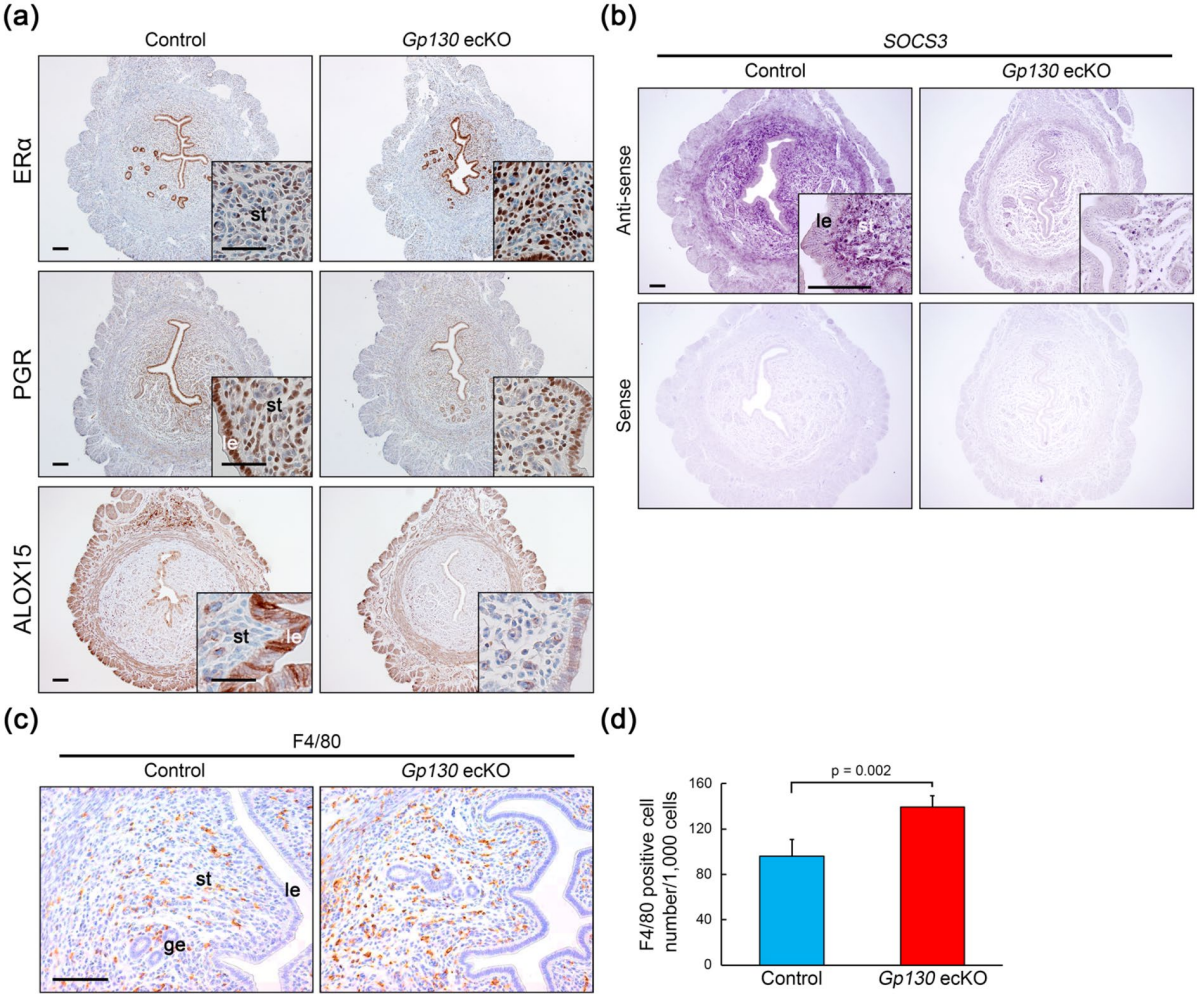
Immunostaining for estrogen- and progesterone-related factors in the control and *Gp130* ecKO uteri. (**a**) Stromal ERα expression is increased in *Gp130* ecKO uteri compared to the control at D4 (1600 h). PGR expression is comparable, but ALOX15 expression, downstream of PGR, is dramatically decreased in the uterine epithelium from *Gp130* ecKO compared to the control. (**b**) *Socs3* mRNA expression (dark purple in anti-sense probe) by ISH on D4 (1600 h). The sense probe shows a background signal. (**c**,**d**) The number of cells positive for F4/80, a marker of macrophages, is significantly increased in *Gp130* ecKO stroma compared to the control at D4. Scale bars: (**a**) 100 µm (with lower magnification) and 50 µm (with higher magnification), (**b**) 100 µm, and (**c**) 50 µm. *le* luminal epithelium, *ge* glandular epithelium, *st* stroma.

In immunohistochemistry (IHC) on day 4 uterine tissues, we confirmed that estrogen receptor α (ERα)-positive cells were increased in the subepithelial stroma, but not in epithelial cells, in *Gp130* ecKO (Fig. 5a). Progesterone receptor (PGR) expression was comparable between control and *Gp130* ecKO (Fig. 5a); however, ALOX15, a known downstream target of the PGR, was significantly decreased in the uterine epithelium in *Gp130* ecKO compared to the control (Fig. 5a). We further confirmed that *Socs3*, which mediates negative feedback regulation by associating with phosphorylated Y759 of Gp130^27^, was markedly reduced in stromal cells by ISH (Fig. 5b). Since SOCS3 acts as an inhibitory factor for granulocyte colony-stimulating factor (G-CSF), a growth factor for neutrophils^28^, its downregulation in the whole endometrium may lead to excessive immune responses. We counted the macrophages by staining of F4/80 and found that F4/80-positive cells were significantly increased in *Gp130* ecKO (P = 0.002) compared to the control (Fig. 5c,d).

### The failure of epithelial remodeling in Gp130 ecKO

**Figure 6:**
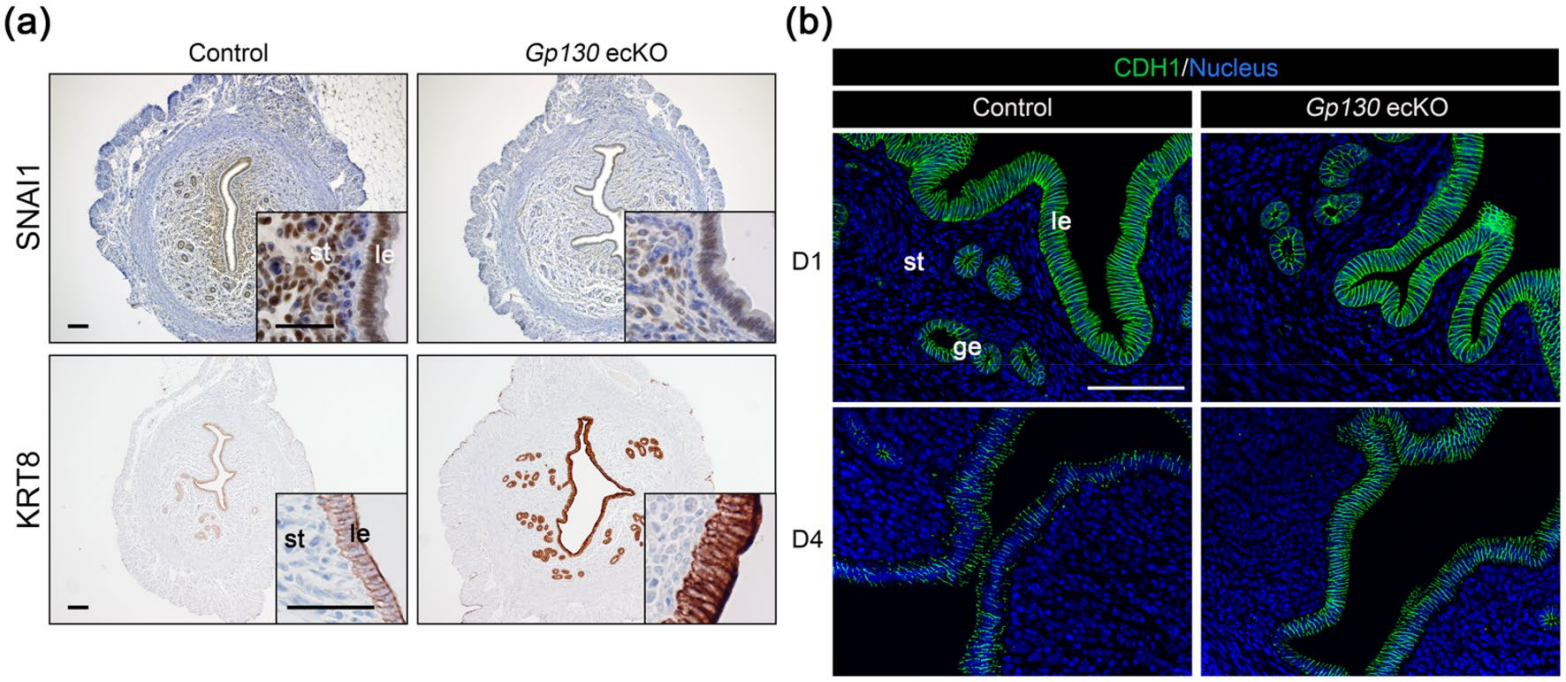
Epithelial remodeling is not altered in *Gp130* ecKO. (**a**) SNAI1 expression was induced in the control uterus at D4 (1600 h) concomitant with decreased KRT8, but not in *Gp130* ecKO. (**b**) CDH1 expression is altered from D1 to D4 (1600 h) in the control but not in *Gp130* ecKO. Scale bars: (**a**) 100 µm (with lower magnification) and 50 µm (with higher magnification), and (**b**) 100 µm. *le* luminal epithelium, *ge* glandular epithelium, *st* stroma.

Our results from *Gp130* ecKO indicated that the abnormal epithelial proliferation observed in uterine-specific *Gp130* knockout females^8^ was not a major reason for the failure of embryo attachment. We investigated the expression of a cell surface mucin, MUC1, which has anti-adhesive properties in the nonreceptive endometrium^29^ by IHC, but there was no significant difference between the control and *Gp130* ecKO (Supplementary Fig. 5). Thus, we further focused on the epithelial remodeling factor, SNAI1, which is transcriptionally regulated by STAT3^30,31^ and represses the epithelial cytoskeleton and cell adhesion molecules, including cytokeratin 8 (KRT8)^32–35^ and E-cadherin (CDH1)^36^. Although we did not detect differences in the expression level of *Snai1* in the endometrium between the control and *Gp130* ecKO by RNA-Seq (Supplementary Table 2), we observed that there were significantly fewer SNAI1-positive cells in *Gp130* ecKO in both epithelium and stroma compared to the control, in which SNAI1-positive cells were widely observed in the uterine tissues (Fig. 6a). Consistent with decreased SNAI1 expression, the expression levels of KRT8 and CDH1 were higher in the endometrial epithelium of *Gp130* ecKO than in that of the control (Fig. 6a,b). On day 4, CDH1 expression was decreased at the cell border in the control, while it was strongly expressed at the cell-to-cell border in *Gp130* ecKO (Fig. 6b). These results indicate that SNAI1 regulated by LIF-Gp130‒mediated signaling contributes to epithelial remodeling for embryo attachment.

## Discussion

It is widely accepted that secreted glandular LIF acts on the epithelial LIFR/Gp130 heterodimer^37^. Previous studies have shown that LIF expression is biphasic; it is expressed in the glandular epithelium in the morning (0800–1000 a.m.) of day 4 and subsequently in the subepithelial stroma surrounding the blastocyst at midnight of day 4^8,11^. On the other hand, LIFR and Gp130 co-localize in the luminal epithelium at midnight of day 4^6^. In particular, Gp130 is dominantly expressed in the glandular epithelium but not in the luminal epithelium at the moment when the glandular LIF is expressed (Fig. 1)^12^. Phosphorylation of STAT3 (pSTAT3) in the luminal epithelium was found at 1600 h of day 4 in the control, but it was absent in *Gp130* ecKO (Fig. 3d), suggesting that an undetectable level of Gp130 in the luminal epithelium may act as a receptor complex, or that LIF transduces signals by binding to a receptor complex other than the LIFR/Gp130 heterodimer, including low-affinity LIFR^38,39^. The observation that pSTAT3 was detectable in the luminal epithelium from wildtype female at day 1 of pregnancy, when the Gp130 is low, might support these possibilities (Supplementary Fig. 6).

The present study demonstrated that the absence of uterine epithelial Gp130 leads to infertility for a reason different from that shown in the uterine-specific *Gp130* knockout^8^. Our results indicated that *Gp130* ecKO cannot support embryo attachment due to the failure of epithelial remodeling caused by the dysregulation of hormone-responsive factors. In fact, stromal ERα was substantially increased and P4-target epithelial genes, including *Alox15* and *Areg*, were dramatically decreased in *Gp130* ecKO (Fig. 4b). AlOX15 oxidizes polyunsaturated fatty acids to generate bioactive lipid metabolites and may play an important role in inflammatory response during implantation^40^. Arachidonic acid metabolites of ALOX15 stimulates various signaling pathways, including PI3K-Akt and MAPK, leading to activate EGR1^40^. EGR1 has been reported as a transcription factor and a key mediator of E2 to control P4-PGR signaling for successful embryo implantation^41^. Loss of EGR1 expression in *Gp130* ecKO is likely to induce imbalance of E2 and P4 function. Areg, member of the epidermal growth factor family, is accumulated in the luminal epithelium of implantation sites prior to blastocyst attachment, overlapping the expression of Ereg and HB-EGF that share EGFR^7,19^. Not only *Areg*, but also *Ereg*, expression was significantly reduced in *Gp130* ecKO uteri, suggesting that these factors are essential for epithelial remodeling and stromal differentiation toward embryo implantation.

Excessive E2 responses and/or decreased P4 responses were found in the disruption of LIF-LIFR/Gp130-STAT3 signal in the endometrium as reported in uterine-specific *Stat3*-deficient^8,9^ and *Gp130*-deficient females^8^, in which abnormal epithelial cell proliferation was manifested during embryo implantation. Epithelial cell proliferation is controlled in a paracrine manner; E2 promotes epithelial cell proliferation by secreting growth factors, such as fibroblast growth factors (FGFs) and insulin-like growth factor (IGF), through stromal ERα^42,43^. Ovulatory E2 promotes epithelial cell proliferation, but P4 secreted from the corpus luteum after coitus counteracts E2 action and suppresses cell proliferation via the epithelial PGR-Indian hedgehog (IHH) signal^44^ and the stromal PGR-Heart and neural crest derivatives expressed 2 (HAND2) signal^17^ prior to embryo implantation. Thus, the attenuation of estrogen-mediated epithelial proliferation is essential for successful implantation. While the uterine-specific *Gp130* knockout utilized by *Pgr*^*Cre*^ showed abnormally sustained cell proliferation in the uterine epithelium during implantation^8^, the uterine epithelium-specific *Gp130* knockout utilized by *Ltf*^*iCre*^ in the present study showed the controlled epithelial cell proliferation; nevertheless, both strains showed embryo attachment failure. These results indicate that cessation of epithelial cell proliferation is necessary but not sufficient to establish embryo implantation and that epithelial Gp130-mediated signaling is not involved in the regulation of epithelial cell proliferation. This is supported by the results of a previous study using the uterine epithelium-specific *Lifr* knockout^45^ and the *Stat3* knockout^9^, in both of which embryo attachment failure was observed without abnormal epithelial cell proliferation during early pregnancy.

In the uterine epithelium-specific *Lifr* knockout, analysis of 3D images led to the conclusion that the failure of crypt formation caused infertility^45^. Those authors analyzed the uterus at day 5 of pregnancy when the early decidualized reaction was started in the wildtype, whereas *Lifr* knockouts failed to achieve an attachment reaction. The presence or absence of the decidual reaction may be responsible for the different crypt formations at day 5 between the *Lifr* knockout and the control in the previous study. Therefore, we checked to see whether embryos were placed in the proper position in the uterine lumen of *Gp130* ecKO at day 4. Although we did not perform 3D imaging analysis, our histological observations suggested that embryos in *Gp130* ecKO were located in the deep antimesometrial side of the slit-like lumen, the same side as in the control. Crypt formation is most likely to be intact even in the loss of epithelial Gp130, suggesting that Gp130-mediated signaling plays an important role in embryo attachment but not in embryo apposition. It is still possible that LIFR and Gp130 have different roles in crypt formation.

In *Gp130* ecKO, an imbalance between E2 and P4 signals was observed, although it did not extend to epithelial growth abnormalities. P4 signaling is absolutely essential for the endometrium to become receptive for the embryo^1,46^, and attenuated P4 signaling resulted in an insufficient decidual reaction^47,48^ and an excessive inflammatory response^49,50^. It was recently reported that female mice with uterine-specific deletion of high-mobility group box-1 (HMGB1) showed severe implantation defects due to decreased P4 signaling concurrent with an excessive immune response, including an increased number of macrophages on day 4 of pregnancy^51^. We found that F4/80-positive macrophages were abnormally sustained in the uterine stroma of *Gp130* ecKO (Fig. 5c,d), suggesting that the absence of epithelial Gp130 attenuated P4-PGR signaling. In addition, we found that the expression of *Socs3*, which suppresses the inflammatory response^52,53^, was dramatically reduced not only in the epithelium but also in the stroma of *Gp130* ecKO compared to the control. These results highlight that epithelial Gp130-mediated signaling is essential to the exertion of appropriate P4-PGR signaling toward embryo implantation. It should be noted that, since *Ltf* is expressed in a subset of immune cells, including neutrophils and macrophages^15,54^, deletion of *Gp130* impacts on their function and inflammation in *Gp130* ecKO. However, macrophage/neutrophil-specific *Gp130* deficiency reduced inflammatory infiltration and delayed and attenuated the disease in the experimental colitis model^54^, suggesting that macrophage infiltration observed in this study is probably due to secondary effect of P4 signal attenuation.

To lose epithelial integrity is one of the major characteristics of a receptive endometrium^55^. It was reported that the conditional deletion of homeobox genes *Msx1*/*Msx2* in the endometrium resulted in embryo attachment failure because of incomplete epithelial alteration^56^. In *Msx1*/*Msx2*-deleted uteri, E-cadherin (coded by *Cdh1*) was more sharply colocalized with β-catenin at the apicolateral border of the luminal epithelium than control uteri^56^. We found that greater epithelial integrity was maintained with dramatically decreased expression of SNAI1, whose transcription is promoted by pSTAT3, in *Gp130* ecKO uteri compared to the control (Fig. 6a). Differential gene expression analysis revealed that integral component of membrane was altered in *Gp130* ecKO, including desmosomal cadherin, desmocollin (Dsc) and desmoglein (Dsg). Our findings demonstrated that Gp130-mediated epithelial remodeling though SNAI1 signaling should be necessary to support embryo attachment. Immunoreaction for SNAI1 was observed in the uterine epithelium and stroma at day 1 of pregnancy and increased nuclear localization was seen at day 4 in wildtype female (Supplementary Fig. 6). Clear nuclear localization of SNAI1 was not observed in the *Gp130* ecKO uteri at day 4, indicating that Gp130-mediated STAT3 activation is necessary to induce SNAI1 localization as reported in the previous study^57^. Interestingly, SNAI1 expression was decreased not only in the epithelium but also in the stroma in *Gp130* ecKO, suggesting that epithelial Gp130-mediated signaling also affects stromal remodeling though SNAI1 signaling.

In summary, our present study suggests that epithelial Gp130-mediated signal, which modulates hormone responses and epithelial remodeling, was essential for successful embryo attachment (Supplementary Fig. 7). The underlying reason of embryo implantation failure in epithelial specific *Gp130*-deficient females was most likely distinct from other studies, such as epithelial specific *Lifr-*deficient females^45^ and uterine specific *Gp130*-defient females^8^. Comparison among different types of genetically engineered implantation failure mouse model provides fundamental insights into LIF-LIFR/Gp130-mediated signal in embryo implantation.

## Materials and methods

All chemicals and reagents were purchased from Sigma-Aldrich (St. Louis, MO, USA) unless otherwise stated. All animal procedures were approved by the Ethical Committee for Vertebrate Experiments at Azabu University (ID#200312-24). All experiments were conducted in accordance with relevant guidelines and regulations, including the Animal Research: Reporting of In Vivo Experiments (ARRIVE) guidelines.

### Animals

Animals were housed in the barrier facility at Azabu University. The following mouse strains were used; *Ltf*^*iCre/*+^ mouse < *Ltf*^*tm1(icre)Tdku*^/J, JAX: 026030 > ^15^ and *Gp130*^*flox/flox*^ (*Gp130f*^*/f*^) mouse < *Il6st*^*tm1Wme*^ >^58^. All strains were maintained on the C57BL/6J background purchased from Jackson Laboratory Japan (Kanagawa, Japan). All mice were fed ad libitum under a 12-h light/12-h dark photocycle at 23 ± 2 °C.

Uterine epithelial-specific *Gp130*-deficient (*Gp130* ecKO) mice were generated by several crossing *Ltf*^*iCre/*+^ strain with *Gp130*^*flox/flox*^ strain. The genotype of mice was confirmed by polymerase chain reaction (PCR) of genomic DNA obtained from tail tissue. The PCR primers used for genotyping were; 5′-GTTTCCTCCTTCTGGGCTCC-3′, 5′-TTTAGTGCCCAGCTTCCCAG-3′ and 5′-CCTGTTGTTCAGCTTGCACC-3′ for *Ltf*^*iCre*^; 5′-GGCTTTTCCTCTGGTTCTTG-3′ and 5′-CAGGAACATTAGGCCAGATG-3′ for *Gp130*^*flox*^. Sexually mature (over 8 weeks old) *Ltf*^*iCre/*+^; *Gp130*^*flox/flox*^ females were used as *Gp130* ecKO and *Ltf*^+*/*+^; *Gp130*^*flox/flox*^ or wildtype females were used as the control. Females were mated with a fertile wildtype male to obtain pregnant samples. The first day of pregnancy was defined as the day when the vaginal plug was first observed. Mice were euthanatized by cervical dislocation after a combination anesthetic with 0.75 mg/kg of medetomidine, 4.0 mg/kg of midazolam, and 5.0 mg/kg of butorphanol. Uterine tissues harvested at indicated day(s) of pregnancy were fixed with 4% paraformaldehyde (PFA) solution in phosphate-buffered saline (PBS) for histological study, fixed with G-Fix (Genostaff, Tokyo, Japan) for in situ hybridization (ISH) or snap-frozen and kept at − 80 °C until used for RNA extraction.

To determine the success of mating, embryos were recovered by flushing the uterus with saline on day 4 or day 5 of pregnancy. The implantation sites were visualized by intravenous injection of 0.1 mL of 1% Chicago blue dye dissolved in saline as previously described^59^ Artificial decidualization was performed by intraluminal injections of 0.02 mL sesame oil after female mice were mated with a vasectomized male as previously described^60^. The wet weight of the oil-infused (stimulated) horn was divided by that of the unstimulated horn.

### Histology

Fixed uterine tissues were paraffin-embedded and paraffin sections (6 µm) were stained with hematoxylin and eosin (H&E). Control and *Gp130* ecKO uterine tissues were placed on the same slide. The depth of the uterine chamber at day 4 of pregnancy was determined by measuring the distance from the mesometrial (M) edge to the antimesometrial (AM) edge of the uterine lumen (M-AM distance) (N ≥ 3). The embryo position was calculated by dividing the distance from the mesometrial edge to the center of the embryo by the M-AM distance. The uterine area was measured using randomly selected uterine cross sections (N = 5 each).

### In situ hybridization (ISH)

ISH was performed as previously described^61^ with some modifications. Briefly, fixed uterine tissues were paraffin-embedded and paraffin sections (6 µm) were mounted on MAS-coated slides (Matsunami Glass Industries, Osaka, Japan) under RNase-free conditions. Sense or antisense digoxigenin (DIG)-labeled RNA probes for *Gp130* (*Il6st*) and suppressor of cytokine signaling 3 (*Socs3*) were purchased from Genostaff. The sections were deparaffinized, rehydrated, and post-fixed in 10% neutral buffered formalin (NBF) for 30 min at 37 °C, followed by the treatment with 0.2% hydrogen chloride and 5 µg/mL proteinase K (FUJIFILM Wako Pure Chemical, Osaka, Japan) for 10 min at 37 °C, respectively. Hybridization was performed with DIG-labeled probes (250 ng/mL) in a humidified chamber at 60 °C overnight. The slides were washed after hybridization, then treated with blocking reagent (Genostaff) for 15 min and alkaline phosphatase-conjugated anti-DIG antibody (1:2000; Roche Diagnostics, Basel, Switzerland) for 1 h at room temperature. The signals were detected by 4-nitro-blue tetrazolium/5-bromo-4-chloro-3-indolyl phosphate (NBT/BCIP, Roche Diagnostics) in a humidified container for 12 h at 4 °C. The sections were counterstained with Kernechtrot solution (Muto Pure Chemicals, Tokyo, Japan). Signals detected by sense probe was used as a control for background levels.

### Immunohistochemistry (IHC) and immunofluorescence (IF)

Fixed tissues were paraffin-embedded and paraffin sections (6 µm) were deparaffinized, hydrated and conducted for antigen retrieval by autoclaving in 10 mM sodium citrate buffer (pH = 6.0) for 5 min. For IHC, the sections were further incubated in 3% hydrogen peroxide diluted with methanol for 10 min. After blocking with a non-specific staining blocking reagent (X0909, DAKO, Carpinteria, CA, USA), the slides were incubated with primary antibodies (shown in Table 1) overnight at 4 °C. For IHC, the slides were followed by the incubation with the Histofine mouse stain kit (Nichirei Biosciences, Tokyo, Japan) for 1 h. Signals were visualized by 3,3′-diaminobenzidine tetrahydrochloride (DAB) and counterstained with hematoxylin. For IF, the slides were incubated with Alexa Fluor 488-conjugated secondary antibodies (Jackson Immuno Research Laboratories, West Grove, PA, USA) for 1 h, and mounted with ProLong Glass Antifade Mountant with NucBlue Stain (P36981, Thermo Fisher Scientific, Waltham, MA, USA). Micrographs were captured by PROVIS AX80 microscopy (Olympus, Tokyo, Japan) or BZ-X700 microscopy (Keyence, Osaka, Japan). All signals were detected under the same lighting conditions between control and *Gp130* ecKO. MKI67 positive epithelial cells were manually counted in randomly selected uterine sections (≥ 100 cells from at least 3 different females in each group).

**Table 1:** Primary antibody list.

Antibody	Species	Source	Catalog No	RRID	Dilution	Application
ERα	Rabbit	Abcam	ab32063	AB_732249	1:200	IHC
PGR	Mouse	Abcam	ab2765	AB_2164316	1:200	IHC
MKI67	Rabbit	Abcam	ab16667	AB_302459	1:200	IHC
MUC1	Rabbit	Abcam	ab15481	AB_301891	1:100	IHC
pSTAT3	Rabbit	Abcam	ab76315	AB_1658549	1:200	IHC
KRT8	Rat	DSHB	TROMA-1	AB_2891089	5 µg/mL	IHC
ALOX15	Mouse	Santa Cruz Biotechnology	sc-133085	AB_2137289	1:200	IHC
SNAI1	Mouse	Santa Cruz Biotechnology	sc-271977	AB_10709902	1:200	IHC
CDH1	Rabbit	Cell Signaling Technology	#3195	AB_2066683	1:1000	IF

*IHC* immunohistochemistry, *IF* immunofluorescence.

### Measurement of serum hormone level

Blood samples from control and *Gp130* ecKO mice were collected on day 4 of pregnancy. Serum was separated by centrifugation (4 °C, 5000×g, 7 min) and stored at − 80 °C until analysis. The serum concentrations of progesterone (P4) and 17β-estradiol (E2) were measured by enzyme-linked immunosorbent assay as described previously^62^.

### RNA extraction and RNA sequence (RNA-seq)

Total RNA was isolated using the RNeasy Plus Mini kit (Qiagen, Venlo, NL). The quality of the RNA was determined by an Agilent 2100 Bioanalyzer (G2939A, Agilent Technologies, Santa Clara, CA, USA). Samples carrying RNA integrity number (RIN) values over 7.0 were used. Sequencing libraries were prepared using the SureSelect Strand-Specific RNA Library Prep kit (G9691A, Agilent Technologies). Briefly, oligo dT magnetic beads were used to purify poly(A) RNA from the total RNA. The libraries were amplified by 13 cycles of polymerase chain reaction (PCR) and then purified with AMPure XP beads (A63880, Beckman Coulter, Brea, CA, USA). The libraries were sequenced using the NextSeq500 system (Illumina, San Diego, CA, USA) with the NextSeq 500/550 High Output Kit v2.5 (75 cycles) at a read length of 75 bp, single-read. FASTQ files were prepared with reads using bcl2fastq ver2.17 (Illumina). The FASTQ files were processed for removal of adaptor sequences, trimmed and quality-based filtered using fastp v0.23.2^63^. The trimmed reads were removed ribosomal RNA using SortMeRNA v4.3.6^64^. The removed reads were mapped onto the reference genome of *Mus musculus* (GRCm39) using STAR v2.7.10a^65^. Mapped reads were counted using RSEM v1.3.3^66^. Differential expression analysis was performed in R v4.2.1 (URL https://www.R-project.org/.) using the EdgeR v 3.38.4 package^67^ and visualized significantly different genes as volcano plot and heatmap using the gplots v3.1.3 and genefilter v1.78.0 packages. GO and pathway analyses were performed in the R software using the clusterProfiler v4.4.4 package^68^ and visualized significantly different GO and pathway as dot plots and a cnet plot.

### Statistical analysis

Statistical analysis was performed by using Student’s *t* test or the Mann‒Whitney U test. All statistical data are shown as mean ± standard error of mean (SEM).

## Supplementary Information

Supplementary Figures.

Supplementary Table S1.

Supplementary Table S2.

## Data Availability

The datasets generated and/or analyzed during the current study are available in the GEO repository, accession number GSE218405.
