# Supplementary material for: Uterine epithelial Gp130 orchestrates hormone response and epithelial remodeling for successful embryo attachment in mice
Source: Sci Rep. 2023 Jan 16;13:854. doi: 10.1038/s41598-023-27859-y (PMC9842754; doi:10.1038/s41598-023-27859-y)
Supplement: Supplementary file 1 — Supplementary Figures. [file 41598_2023_27859_MOESM1_ESM.pdf]

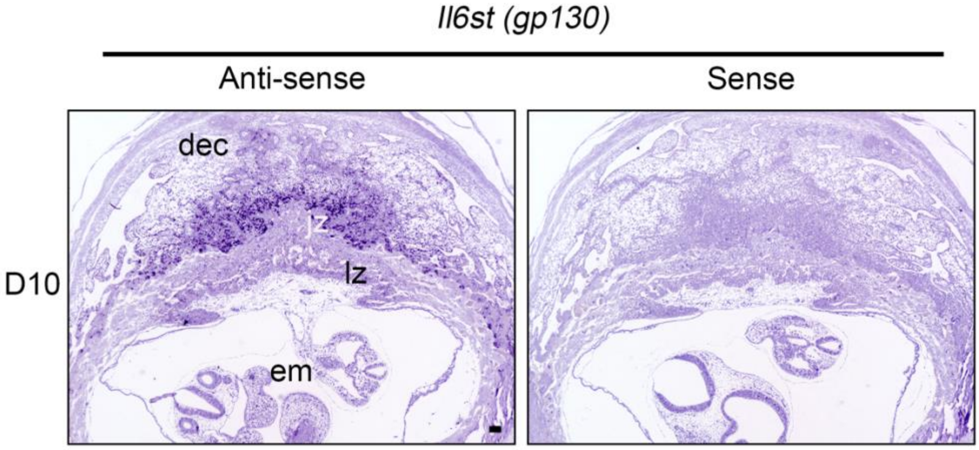

Supplemental Fig.1. *Il6st* (*Gp130)* mRNA expression in the mouse placenta at day 10 (D10) of pregnancy. The sense probe shows background signal. Scale bar, 100 µm. em: embryo; dec: decidua; jz: junctional zone; lz: labyrinth zone.

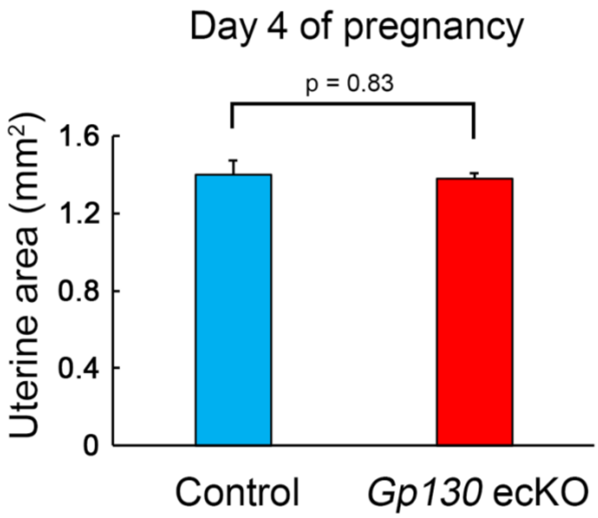

Supplemental Fig.2. Uterine area between the control and *Gp130* ecKO at D4 (1600 h).

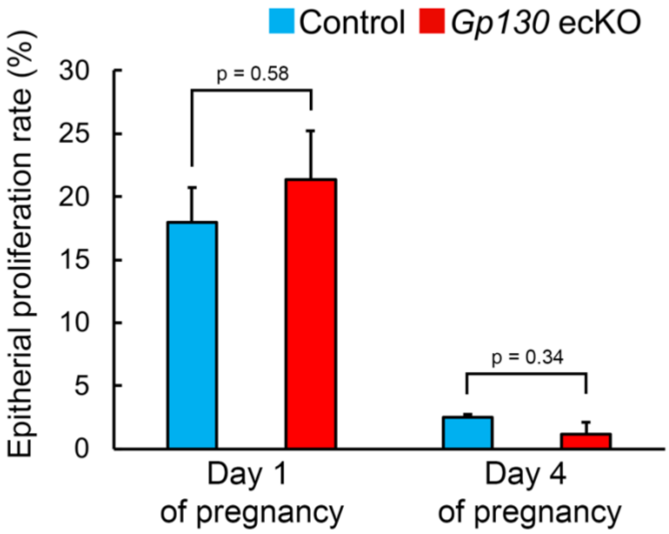

Supplemental Fig.3. Epithelial cell proliferation rate between the control and *Gp130* ecKO at D1 and D4 (1600 h).

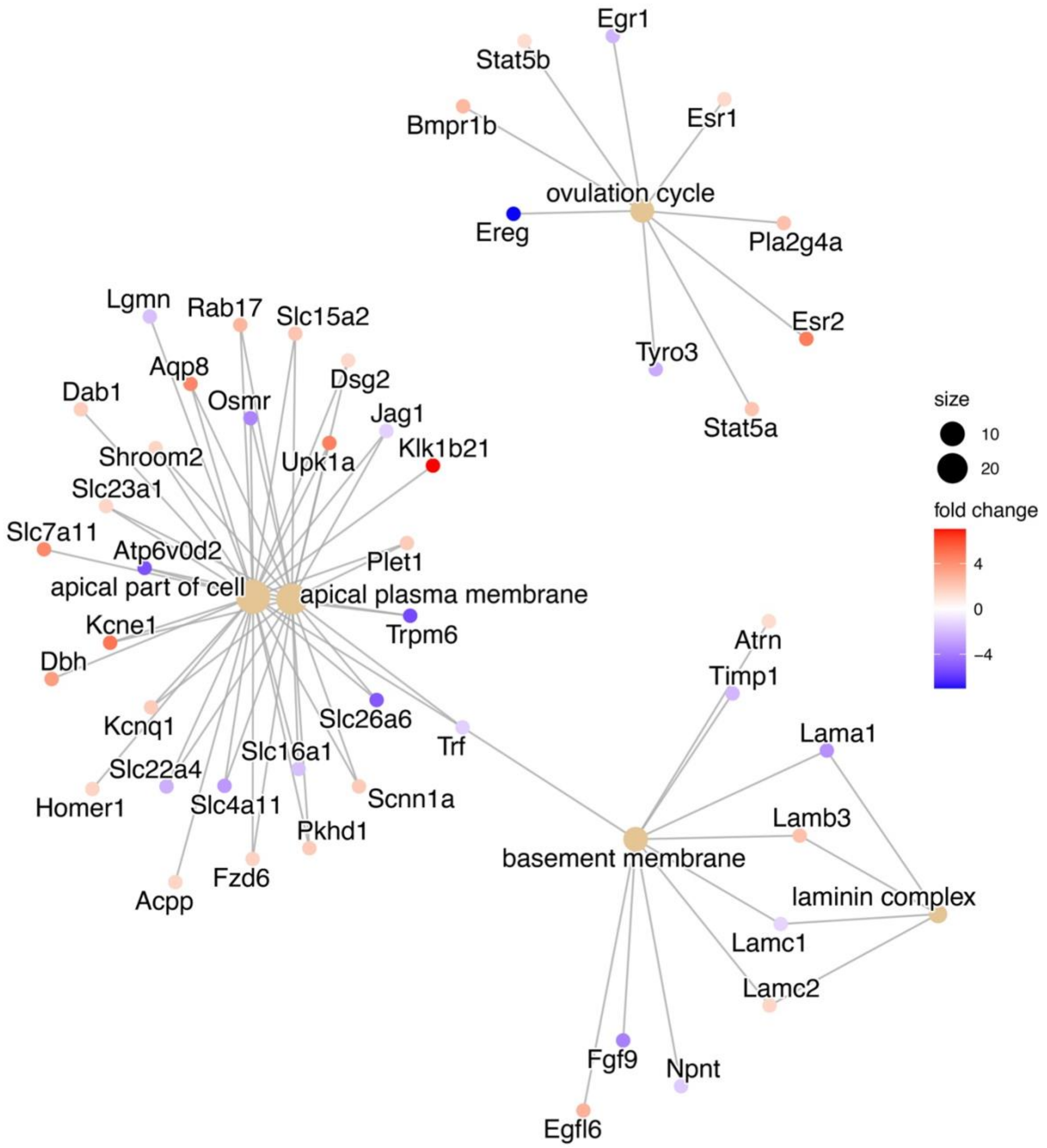

Supplemental Fig.4. Cnet plot of GO analysis in Fig. 4c.

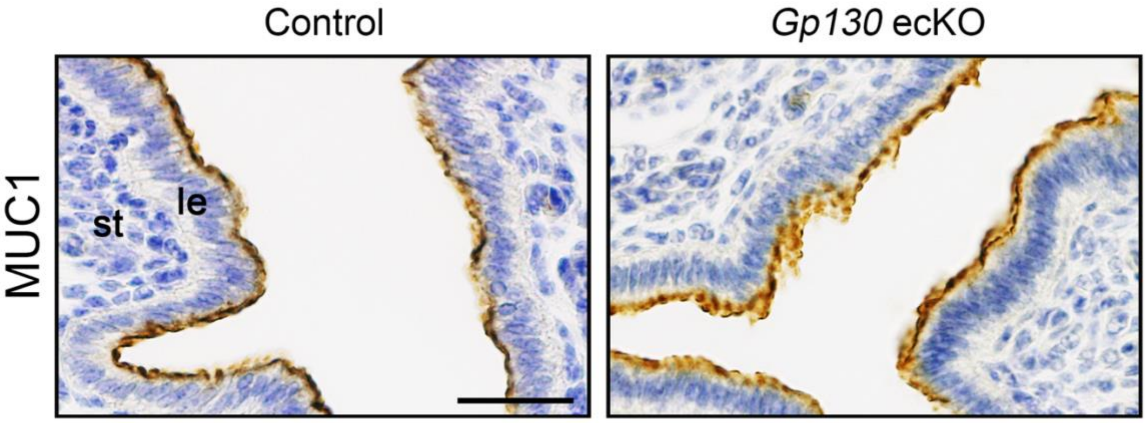

Supplemental Fig.5. MUC1 expression in the uterus from the control and *Gp130* ecKO at D4 (1600 h). Scale bar, 100 μm. le: luminal epithelium; st: stroma.

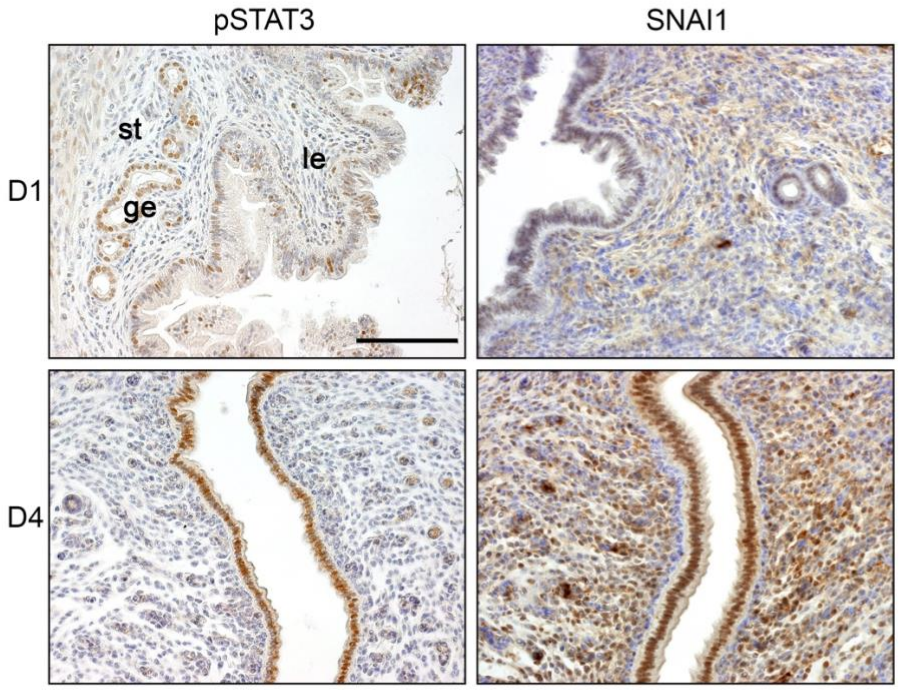

Supplemental Fig.6. IHC for pSTAT3 and SNAI1 in the uterus from the control at D1 and D4. Scale bar, 100 µm. le: luminal epithelium; ge: glandular epithelium; st: stroma.

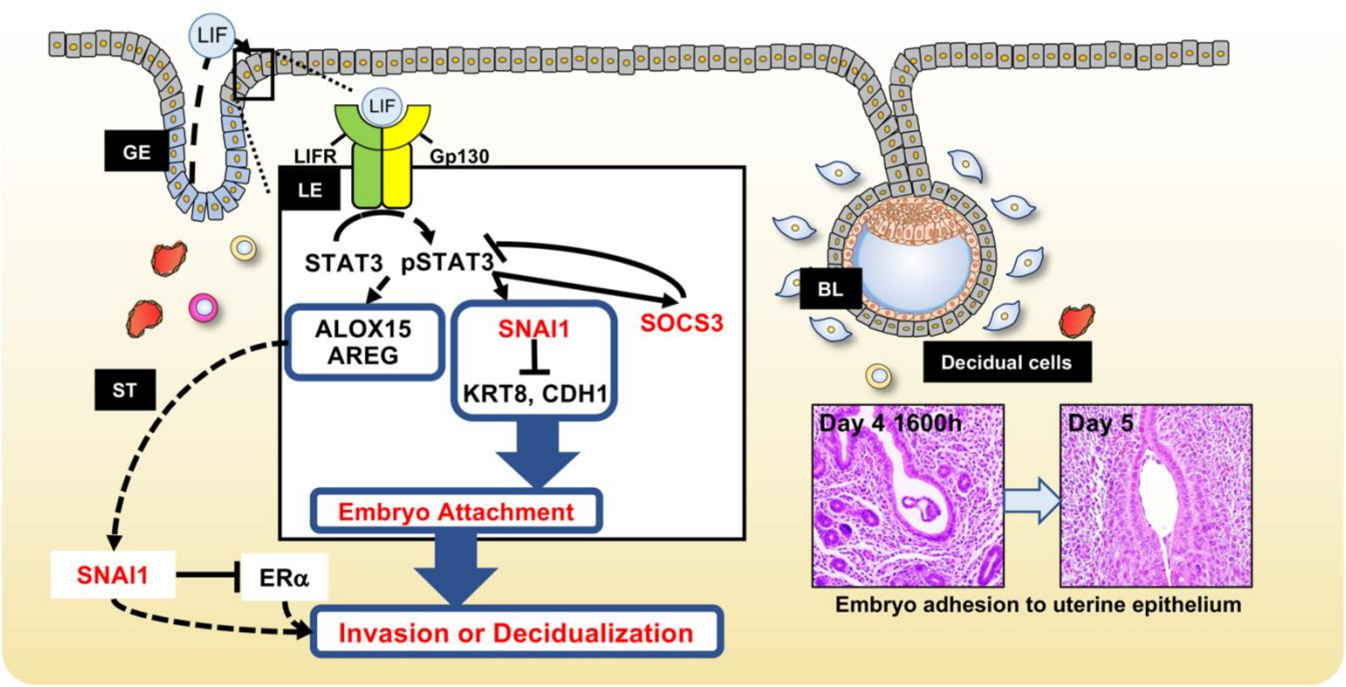

Supplemental Fig.7. Model: Gp130-mediated LIF signaling in the uterus for successful embryo implantation in mice. LE: luminal epithelium; GE: glandular epithelium; ST: stroma; BL: blastocyst.
