# Supplementary material for: Uterine epithelial Gp130 orchestrates hormone response and epithelial remodeling for successful embryo attachment in mice
Source: Sci Rep. 2023 Jan 16;13:854. doi: 10.1038/s41598-023-27859-y (PMC9842754; doi:10.1038/s41598-023-27859-y)
Supplement: Supplementary file 2 — Supplementary Table S1. [file 41598_2023_27859_MOESM2_ESM.pdf]

Supplemental table 1. List of mouse phenotype with altered LIFR/Gp130 – STAT3 signals

Gene	Defective uterine part	Genotype	Embryo implantation	Proliferation of the uterine epithelial cells	STAT3 status in the uterine epithelium	Reference
Stat3	Whole uterus	*Pgr*^{Cre}*-Stat3*	Failure	Sustained	Non-phosphorylated	PMID: 23885093
	Whole uterus	*Pgr*^{Cre}*-Stat3*	Failure	Sustained	ND	PMID: 32968170
	Whole uterus	*Pgr*^{iresCre}*-Stat3*	Failure	ND	ND	PMID: 34402137
	Uterine epithelium	*Wnt7a-Cre-Stat3*	Failure	ND	ND	PMID: 24100212
	Uterine epithelium	*Ltfi*$^{Cre-}$*Stat3*	Failure	Suppressed	Non-phosphorylated	PMID: 32968170
	Stroma	*Amhr2*^{Cre}*-Stat3*	Embryonic resorption	ND	ND	PMID: 24983622
	Stroma	*Amhr2*^{Cre}*-Stat3*	Failure	Sustained	Phosphorylated	PMID: 32968170
Lifr	Uterine epithelium	*Ltf*^{Cre}*-Lifr*	Severe loss of litters	ND	Non-phosphorylated	PMID: 28368537
	Uterine epithelium	*Ltf*^{iCre}*-Lifr*	Failure	Suppressed	Non-phosphorylated	PMID: 34402888
	Stroma	*Amhr2*^{Cre}*-Lifr*	No phenotype	Suppressed	Phosphorylated	PMID: 34402888
Gp130	Whole uterus	*Pgr*^{Cre}*-Gp130*	Failure	Sustained	ND	PMID: 23885093
(*Il6st*)	Whole uterus	*Pgr*^{iresCre}*-Gp130*	Failure	ND	ND	PMID: 34402137
	Uterine epithelium	*Ltf*^{iCre}*-Gp130*	Failure	Suppressed	Non-phosphorylated	Present study

ND, not determined
